# Molecular Docking and Dynamics Simulation Revealed the Potential Inhibitory Activity of ACEIs Against SARS-CoV-2 Targeting the *h*ACE2 Receptor

**DOI:** 10.3389/fchem.2021.661230

**Published:** 2021-05-04

**Authors:** Ahmed A. Al-Karmalawy, Mohammed A. Dahab, Ahmed M. Metwaly, Sameh S. Elhady, Eslam B. Elkaeed, Ibrahim H. Eissa, Khaled M. Darwish

**Affiliations:** ^1^Department of Pharmaceutical Medicinal Chemistry, Faculty of Pharmacy, Horus University-Egypt, Damietta, Egypt; ^2^Pharmaceutical Medicinal Chemistry & Drug Design Department, Faculty of Pharmacy (Boys), Al-Azhar University, Cairo, Egypt; ^3^Pharmacognosy Department, Faculty of Pharmacy (Boys), Al-Azhar University, Cairo, Egypt; ^4^Department of Natural Products, Faculty of Pharmacy, King Abdulaziz University, Jeddah, Saudi Arabia; ^5^Department of Pharmaceutical Sciences, College of Pharmacy, AlMaarefa University, Ad Diriyah, Saudi Arabia; ^6^Department of Pharmaceutical Organic Chemistry, Faculty of Pharmacy (Boys), Al-Azhar University, Cairo, Egypt; ^7^Department of Medicinal Chemistry, Faculty of Pharmacy, Suez Canal University, Ismailia, Egypt

**Keywords:** COVID-19, molecular docking, molecular dynamics, ACEIs, hACE2

## Abstract

The rapid and global spread of a new human coronavirus, Severe Acute Respiratory Syndrome Coronavirus 2 (SARS-CoV-2) has produced an immediate urgency to discover promising targets for the treatment of COVID-19. Here, we consider drug repurposing as an attractive approach that can facilitate the drug discovery process by repurposing existing pharmaceuticals to treat illnesses other than their primary indications. We review current information concerning the global health issue of COVID-19 including promising approved drugs, e.g., human angiotensin-converting enzyme inhibitors (*h*ACEIs). Besides, we describe computational approaches to be used in drug repurposing and highlight examples of *in-silico* studies of drug development efforts against SARS-CoV-2. Alacepril and lisinopril were found to interact with human angiotensin-converting enzyme 2 (*h*ACE2), the host entranceway for SARS-CoV-2 spike protein, through exhibiting the most acceptable rmsd_refine values and the best binding affinity through forming a strong hydrogen bond with Asn90, which is assumed to be essential for the activity, as well as significant extra interactions with other receptor-binding residues. Furthermore, molecular dynamics (MD) simulations followed by calculation of the binding free energy were also carried out for the most promising two ligand-pocket complexes from docking studies (alacepril and lisinopril) to clarify some information on their thermodynamic and dynamic properties and confirm the docking results as well. These results we obtained probably provided an excellent lead candidate for the development of therapeutic drugs against COVID-19. Eventually, animal experiments and accurate clinical trials are needed to confirm the potential preventive and treatment effect of these compounds.

## Introduction

In December 2019, rumors began to spread about the prevalence of a new unknown pneumonia-like illness in Wuhan, the capital of Hubei Province in China. Afterward, on February 11, 2020, the WHO reported a novel coronavirus as the causative agent of clusters of the new illness. Severe Acute Respiratory Syndrome Coronavirus 2 (SARS-CoV-2) or COVID-19 was the name that the WHO designated for the disease caused by the novel coronavirus (Coronaviridae Study Group of the International Committee on Taxonomy of Viruses, 2020). Since the beginning of the outbreak, infections have expanded rapidly into multiple simultaneous epidemics worldwide. As of January 23, 2021, 99,071,240 confirmed COVID-19 cases and 2,124,086 COVID-19-related deaths have been reported across more than 221 countries (Culp, 2021).

The COVID-19 with influenza-like symptoms ranging from mild discomfort to severe lung injury and multi-organ failure, eventually leading to death (Rothe et al., 2020). Effective treatments for SARS-CoV-2 infection do not currently exist. Thus, it will be of great benefit to identify and repurpose already well-characterized compounds and approved drugs for use in combating COVID-19 (https://www.who.int/emergencies/diseases/novel-coronavirus-2019).

Drug repurposing or drug reprofiling is a promising field in drug discovery for identifying new therapeutic uses for already studied drugs (Khattab and Al-Karmalawy, 2021; Khattab et al., 2021). These drugs could be either currently approved and marketed for another use or withdrawn because of adverse effects (Ashburn and Thor, 2004). Available clinical trials at ClinicalTrials.gov (https://clinicaltrials.gov/) include the investigation of previously approved drugs for different indications, e.g.,: telmisartan and losartan. It offers a great opportunity to the traditional *de novo* drug discovery since the success rate of developing a new molecular entity is 2.01% only, and the number of approved drugs has been declining since the 1990's (Yeu et al., 2015). In the last decade, about one-third of the approvals correspond to drug repurposing, and repurposed drugs currently generate around 25% of the annual revenue for the pharmaceutical industry (Talevi and Bellera, 2020). As examples of the most common treatment, hydroxychloroquine, an antimalarial agent with anti-inflammatory and immunomodulatory activities, has shown inhibitory activity for SARS-CoV-2 similar to previous studies on SARS-CoV-1 (Sanders et al., 2020). It has been investigated for use by COVID-19 patients based on positive *in vitro* and limited clinical data. Also, azithromycin, a macrolide antibiotic, was found to raise the efficacy of hydroxychloroquine as a complementary therapy (Lover, 2020).

Computer-aided drug discovery is one of the most important approaches to investigate the activity of a drug through computational structure-based drug discovery. Different software tested the interaction between the tested compounds and the binding site through physics-based equations used to calculate their binding affinities (Sliwoski et al., 2014). SARS-CoV-2 proteins, particularly proteases and spike proteins (Prajapat et al., 2020), have been targeted in many docking investigations hoping to understand the key amino acids essential for the interactions at the active site in SARS-CoV-2 (Calligari et al., 2020; Dahab et al., 2020; Khan et al., 2020; Kumar et al., 2020; Mohammad et al., 2020; Wu et al., 2020; Jairajpuri et al., 2021).

In general, various organ systems are believed to participate in COVID-19 due to the widespread expression of the primary SARS-CoV-2 entry receptor, human angiotensin-converting enzyme 2 (*h*ACE2) (Groß et al., 2020). Angiotensinogen (AGT) as a key substrate of the Renin-Angiotensin System (RAS) is mainly synthesized by the liver and is cleaved by renin to form Ang I (proangiotensin). In the pulmonary circulation, Ang I is easily activated to *h*ACE2 (Wu et al., 2018). ACE is a zinc metallopeptidase ectoenzyme predominantly found in the lungs and was originally isolated in 1956 as (hypertension converting enzyme) (Skeggs et al., 1955). In 2000, genomic-based strategies led to the discovery of *h*ACE2, a human ACE homolog. *h*ACE2 receptors which are the door through which the virus enters into cells and also the conductor of several pathophysiological reactions associated with the clinical features of the disease, with potential therapeutic implications (Donoghue et al., 2000).

Taking into account the characteristics of the mode of entry of this coronavirus to human cells through binding with *h*ACE2 and extensive scientific and clinical evidence information on the RAS, the hypothesis of the involvement of this system in the pathophysiology of COVID-19 was born (Gurwitz, 2020). The SARS-CoV-2 virus enters the airway and binds, utilizing the S (Spike) protein on its surface, to the membrane protein *h*ACE2 in type 2 alveolar cells. The S protein-*h*ACE2 complex is internalized by endocytosis and facilitates the entry of each virion into the cytoplasm (Wan et al., 2020).

*h*ACE2 is involved in modulating blood pressure and establishing blood pressure homeostasis. Recently, a debatable question has risen, whether using antihypertensive medications will have a favorable impact on people infected with SARS-CoV-2 or a deleterious one, mainly since ACEIs and ARBs therapy can modulate the expression of *h*ACE2protein (Vaduganathan et al., 2020).

We suppose that inhibition of the *h*ACE2 catalytic pocket by small molecules, e.g., ACEIs, could change the conformation of *h*ACE2 in such a way that it could block SARS-CoV-2 entry inside host cells through *h*ACE2 (Du et al., 2009).

Recently, a new promising success was reported: a group of scientists claimed that human recombinant soluble ACE2 (*hrs*ACE2) can block the early stages of SARS-CoV-2 infections (Monteil et al., 2020). Moreover, telmisartan (ClinicalTrials.gov ID: NCT04355936) and losartan (ClinicalTrials.gov ID: NCT04312009) were proposed as alternative options for treating COVID-19 patients before the development of acute respiratory distress syndrome (ARDS) (Alnajjar et al., 2020; Gurwitz, 2020).

Interestingly, Zhang et al. found that among patients with hypertension hospitalized with COVID-19, inpatient treatment with ACEIs or angiotensin receptor blockers (ARBs) was associated with a lower risk of all-cause mortality compared with ACEI/ARB non-users (Zhang et al., 2020). Also, ACEIs proved to be particularly beneficial not only in controlling high blood pressure but also in reducing the incidence of stroke, by downregulating tissue factor synthesis in monocytes (Dézsi, 2000; Napoleone et al., 2000).

**Figure 1:**
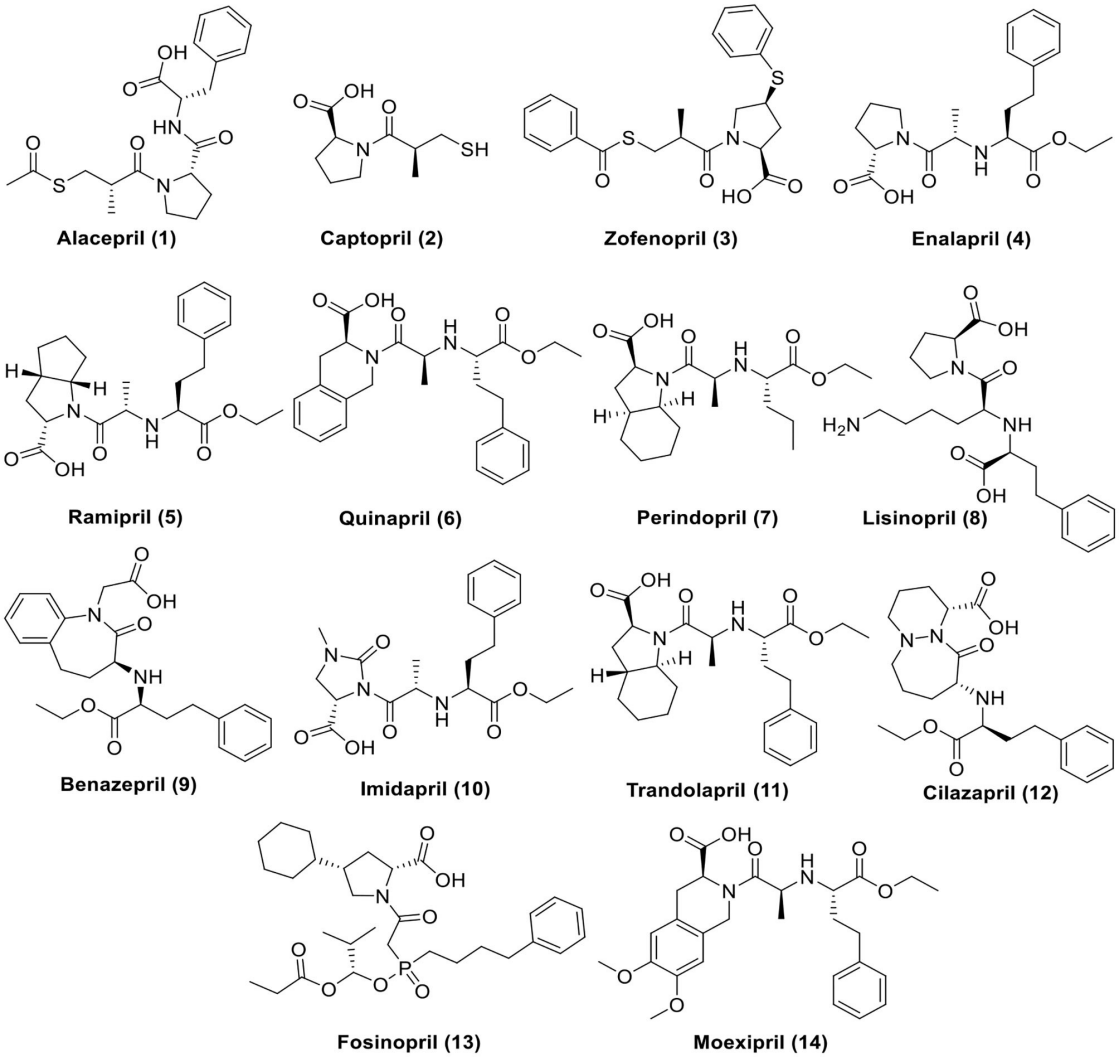
Chemical structures of the tested ACEIs.

**Figure 2:**
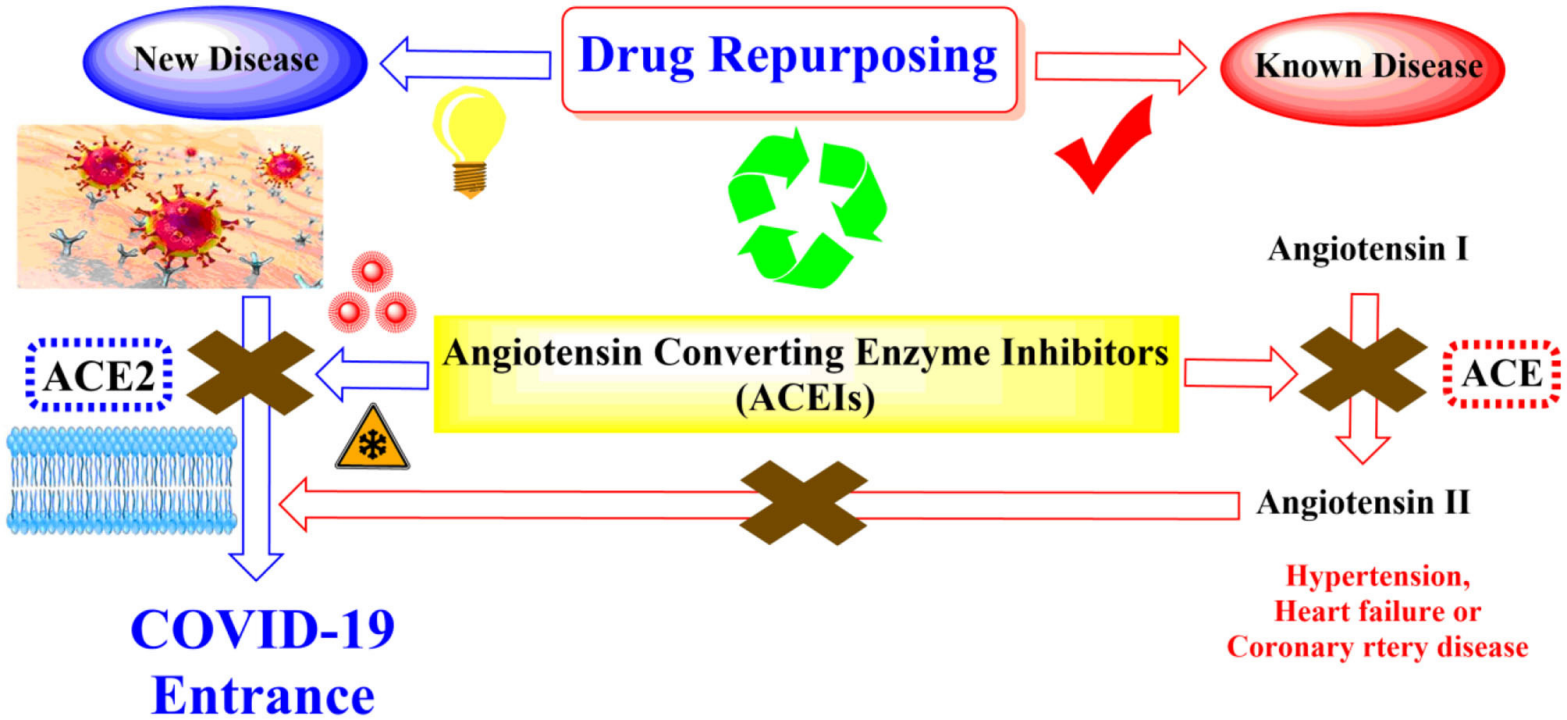
Schematic representation showing the idea of repurposing the FDA-approved ACEIs as COVID-19 entrance inhibitors through the inhibition of the *hrs*ACE2 receptor.

For these reasons and in continuation to our previous works targeting SARS-CoV-2 (Alnajjar et al., 2020; Zaki et al., 2020; Al-Karmalawy et al., in press), the authors present a promising computational study including molecular docking and dynamics simulation for almost all FDA approved members of ACEIs (Figure 1) against the receptor-binding domain (RBD) of the spike protein of SARS-CoV-2 in complex with *h*ACE2 hoping to repurpose them effectively for the potential treatment of COVID-19 infection. However, we propose that ACEIs having the ability to block the *hrs*ACE2 receptor and so prevent the entrance of SARS-CoV-2 through its spike protein (Figure 2). Collectively, the main aim of the study is to investigate the potentiality of ACEIs, as promising small ligand molecules with drug-likeness properties, to accommodate the *N*-acetyl-β-glucosamine (NAG) specific binding site at the *h*ACE2 protein target. Accommodation of such pocket could permit distrusted glycan stability within such site being at proximity to the *h*ACE2/SARS-CoV-2 Spike protein receptor-binding domain (RBD) interface. Accommodating this site by small molecules may impact the SARS-CoV-2 Spike protein owing to the reported findings of the glycan-mediated influence/interference with the *h*ACE2/SARS-CoV-2 Spike protein association as well as spike epitopic recognition (Li et al., 2005; Banerjee et al., 2020; de Andrade et al., 2020; Devaux et al., 2020; Grant et al., 2020). Therefore, the affinity of ACEIs against the *h*ACE2-NAG binding site was investigating through molecular docking and dynamics studies having the glycan NAG as a competitor binder and reference ligand.

## Materials and Methods

Both the molecular docking studies using MOE 2014.09 suite (Vilar et al., 2008) and molecular dynamics simulation using the GROMACS-2019 software package and CHARMM36 force field (da Silva et al., 2020) were applied in this study.

### Molecular Docking Studies

To find a potential candidate for treating COVID-19, molecular docking studies were performed over 14 ACEIs on the binding pocket of the SARS-CoV-2 chimeric receptor-binding domain complexed with its receptor human *h*ACE2 (PDB IDs: 6VW1) (Shang et al., 2020). The chemical structures of drugs tested for docking study are depicted in Figure 1. The co-crystallized ligand *N*-Acetyl-*D*-Glucosamine (NAG) was used as a reference standard.

The tested compounds were sketched using ChemDraw 2014, imported into MOE, and subjected to 3D protonation and energy minimization up to 0.01 gradient. Then the co-crystallized ligand (NAG) and the tested compounds were imported into the same database and saved in the form of an MDB file to be used in the docking calculations with SARS-CoV-2 spike protein, 6VW1. The crystal structure was obtained from Protein Data Bank (http://www.rscb.org) with good resolutions 2.68 Å (Shang et al., 2020). The crystal structures were prepared following the detailed procedure described earlier (Al-Karmalawy and Khattab, 2020; Ghanem et al., 2020). They were imported into MOE and the structure preparation wizard of MOE was used to correct all the issues in protein structures. The hydrogen atoms were added to structures in their standard geometry, and all solvent molecules were removed from the structures then subjected to energy minimization. The final optimized structures were saved in the working directory. Triangle matcher and refinement methods were used for performing docking studies. Rigid receptor as refinement methodology and GBVI/WSA dG as the scoring methodology for selection of the best 20 poses from 100 different poses for each tested compound. The scoring methods were adjusted to their default values (Samra et al., 2021). After completion of docking processes, the obtained poses were studied and the best ones showing the best acceptable rmsd_refine values with the same binding mode of the native ligand were selected. Also, a program validation process was performed at first and confirmed by a low RMSD value (<1Å) as described before (Eliaa et al., 2020).

### Molecular Dynamics Simulation

The best-docking scored models of the most promising leads, alacepril and lisinopril, in complex with *h*ACE2 protein were chosen as starting coordinates for 100 ns all-atom molecular dynamics simulation using a GROMACS-2019 software package (GNU, General Public License; http://www.gromacs.org) and CHARMM36 force field (da Silva et al., 2020). Each ligand–protein complex was solvated within a cubic box of the transferable intermolecular potential with a three-points (TIP3P) water model (100 × 100 × 100 Å) allowing a minimum of 10 Å marginal distance between protein and each side of the 3D box (Izadi et al., 2014). The CHARMM force field parameters for the investigated ligands were automatically generated using the CHARMM General Force Field (CGenFF) program (Vanommeslaeghe et al., 2009) (ParamChem project; https://cgenff.umaryland.edu/). Under periodic boundary conditions implementation, the protein residues were assigned for their standard ionization states at physiological conditions (pH 7.0), and the whole complexes were neutralized via sufficient numbers of K^+^ and Cl^−^ ions added via Monte-Carlo ion-placing method (Ross et al., 2019). The MD simulation was conducted over three stages and 1,000 kJ/mol.nm^2^ force constant was used for restraining all heavy atoms and preserving original protein folding (Helal et al., 2020). The first stage involved initial optimization of each system geometry using 5,000 iterations (5 ps) with the steepest descent algorithm. The subsequent step involved system two-staged equilibration where the system was conditioned for 100,000 iterations (100 ps) at each stage. The first equilibration stage was proceeded under constant Number of particles, Volume, and Temperature (NVT) ensemble guided by the Berendsen temperature coupling method for regulating the temperature within the 3D box (Golo and Shaitan, 2002). Subsequently, the second equilibration stage was performed under a constant Number of particles, Pressure, and Temperature (NPT) ensemble at 1 atm and 303.15 K guided by using the Parrinello-Rahman barostat (Tuble et al., 2004).

Finally, the MD simulations were run for 100 ns under constant pressure (NPT ensemble) and long-range electrostatic interactions were computed using Particle Mesh Ewald (PME) algorithm (Darden et al., 1998). Adopting such a highly accurate and rapid algorithm for treating long-range Coulomb interactions to achieve stable nanosecond trajectories within highly polar biomolecules like proteins. However, the implemented linear constraint LINCS method was used to constrain all covalent bond lengths, including hydrogens, allowing an integration time step size of 2 fs (Hess et al., 1997). The non-bounded interactions, Coulomb (electrostatic potential), and Lennard Jones (Pauli repulsion and hydrophobic/van der Waals attractions) interactions were truncated at 10 Å using the Verlet cut-off scheme (Páll and Hess, 2013). Throughout the MD simulation, the CHARMM36m all-atom force field was applied for both the ions and protein (Best et al., 2013). Computing comparative data, including RMSD and radius of gyration (Rg), was performed through analyzing the MD trajectories using the GROMACS built-in tools. Moreover, the Distance Calculation Tool, at Visual Molecular Dynamics 1.9.3 (VMD) package (the University of Illinois at Urbana-Champaign, USA), was utilized to calculate the change in the distance between the specified ligand/protein atoms over the whole simulation period (Humphrey et al., 1996). Such an approach permitted monitoring and investigating the possibility of interactions of ligands with the most important protein residues. Finally, the binding-free energy between the ligand and protein was estimated via the GROMACS “*g_mmpbsa*” module (Kumari et al., 2014). The Pymol graphical software ver. 2.0.6 (Schrödinger^TM^, NY, USA) was utilized for figure generation of ligand–protein conformational analysis (Delano, 2002).

## Results and Discussion

### Molecular Docking Studies

**Figure 3:**
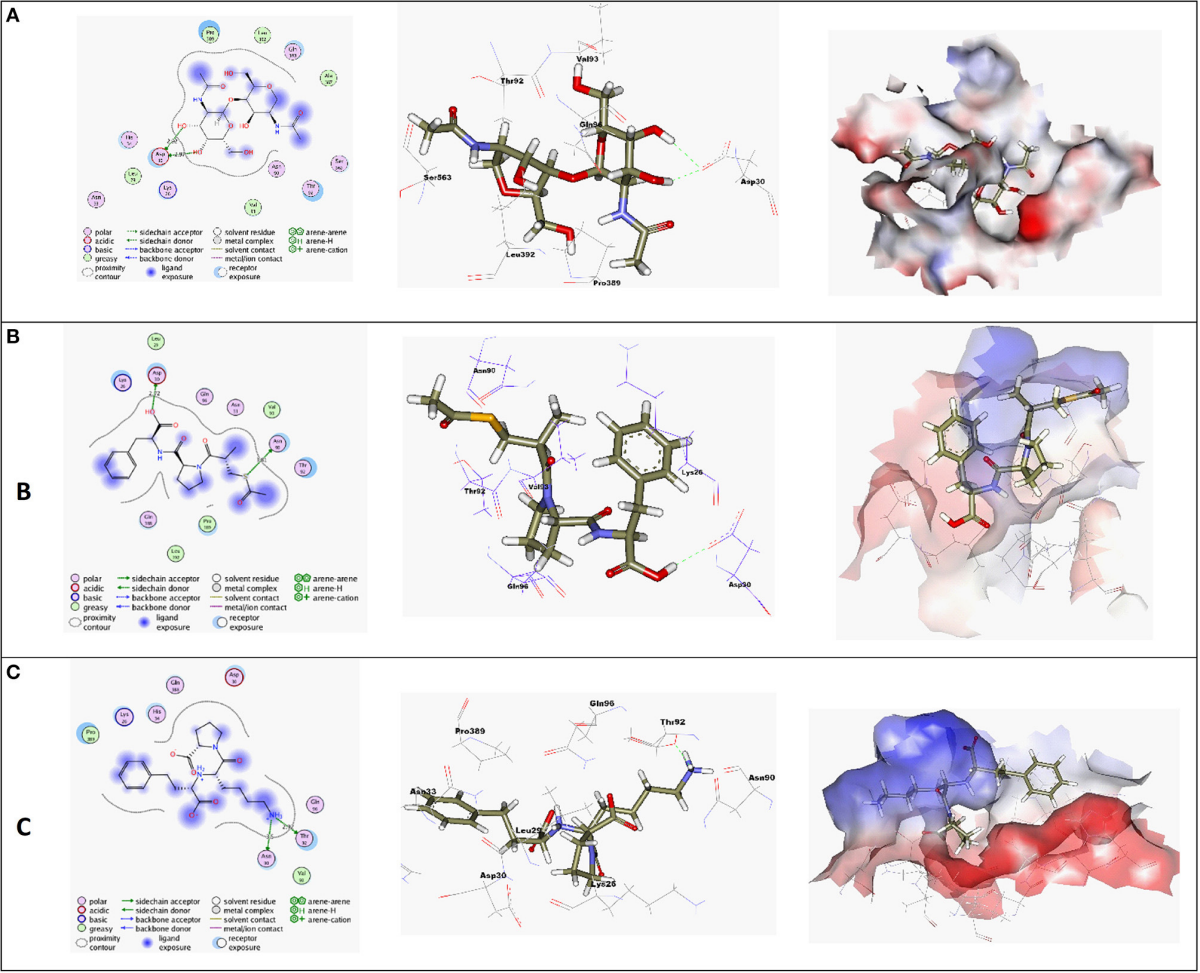
**(A)** High-resolution crystal structures of coronavirus target explain the native ligand (NAG) in the active pocket (PDB ID: 6VW1, Score = −4.4, RMSD = 1.3). **(B)** High-resolution crystal structures of coronavirus target explain Alacepril in the active pocket (PDB ID: 6VW1, Score = −5.1, RMSD = 1.3). **(C)** High-resolution crystal structures of coronavirus target explain Lisinopril in the active pocket (PDB ID: 6VW1, Score = −4.6, RMSD = 1.3). N.B: The surface and maps representations show the H-bond donor, H-bond acceptor, and hydrophobic regions around the docked compound.

Molecular docking simulations were performed in order to investigate the potentiality of small drug-like molecules, like ACEIs, to engage the *h*ACE2 glycosylated site and/or vicinal cavity in a way that would disrupt the glycosylation process of the *h*ACE2, leading to the modulation of *h*ACE2-RBD interactions. Actually, this crystallized *N*-glycan is covalently linked to the aimed nitrogen of the asparagine residue of the protein. Nevertheless, the approach of *N*-glycan and its existence within the pocket is highly guided by both Coulomb's electrostatic interactions and Lenard-Johns van der Waal potential energy with different target residues comprising the *h*ACE2 pocket lining. In these regards, this *N*-glycan was considered as a reference ligand to investigate the ability of the investigated ACEIs to compete with it for engaging this glycosylated site and vicinal cavity. Throughout the adopted docking protocol, this *N*-glycan binder was fitted inside the binding pocket of SARS-CoV-2 spike protein showing one hydrogen bond with Asn90 (2.84 Å, binding score = −4.4, RMSD = 1.3), Figure 3A.

A molecular docking simulation of the target compounds and the native ligand into the spike protein active site was carried out. Many poses were obtained with better binding modes and interactions inside the receptor pocket. The poses with the most acceptable rmsd_refine values (related to the closeness of the selected pose to the original ligand position inside the receptor pocket) and the same binding mode of the ligand were selected. Results of energies and different interactions with amino acids of the spike protein pocket are shown in Table 1. They got stabilized at the binding site of spike protein by variable several electrostatic bonds.

**Table 1:** Receptor interactions and binding energies of ACEIs drugs and NAG inhibitor into the spike protein of SARS-CoV-2.

**No**.	**ACEIs**	**S^**a**^ Kcal/mole**	**RMSD_Refine^**b**^**	**Amino acid bond**	**Distance Å**
1	Alacepril	−5.10	1.3	Asn90/H-acceptor	3.81
				Asp30/H-acceptor	2.72
2	Captopril	−3.40	1.4	Asp30/H-acceptor	3.76
3	Zofenopril	−4.6	1.6	Pro389/arene-H	4.34
4	Enalapril	−4.8	1.5	Asp30/H-donor	2.94
				Asp30/H-donor	2.94
5	Ramipril	−4.6	1.7	Lys26/H-acceptor	4.29
				Lys26/H-acceptor	3.98
6	Quinapril	−4.60	1.7	Pro389/arene-H	4.52
				Gln96/H- acceptor	3.07
7	Perindopril	−4.2	1.7	Asp30/H-donor	3.31
				Asp30/H-donor	3.32
				Asp30/H- acceptor	3.31
				Asp30/H- acceptor	3.32
8	Lisinopril	−4.70	1.3	Asn90/H-acceptor	3.5
				Thr92/H-acceptor	2.92
9	Benazepril	−4.70	1.3	Lys25/H-donor	3.07
				Lys25/H-donor	3.07
10	Imidapril	−4.4	1.8	Asp30/H-donor	3.45
				Asp30/H-donor	3.45
11	Trandolapril	−5.60	1.2	Asp30/H-donor	2.75
				Asp30/H-donor	2.75
				Gln95/H-acceptor	2.91
12	Cilazapril	−4.5	1.6	Pro389/arene-H	4.49
				Asp30/H- donor	3.24
				Asp30/H- donor	3.24
				Asp30/H- donor	3.60
13	Fosinopril	−5.04	1.7	Gln96/H-acceptor	4.36
14	Moexipril	−5.10	1.7	Asp30/H- donor	4.25
				Asp30/H- donor	3.16
				Asp30/H- donor	3.36
15	NAG	−4.4	1.3	Asp30/H- donor	2.97
				Asp30/H- donor	2.92

a *S: the score of placement of a compound into the binding pocket of protein using London dG scoring function*.

b *RMSD_Refine: the root-mean-squared-deviation (RMSD) between the heavy atoms of the predicted pose (after refinement) and those of the crystal structure (before refinement)*.

Most compounds showed acceptable RMSD values close to the NAG inhibitor, but only alacepril and lisinopril have the same binding mode of the NAG. For alacepril, binding interactions with 6VW1 (binding score = −5.1, RMSD = 1.3) are given in Figure 3B, two hydrogen bonds were recorded, one of them with Asn90 (3.81 Å), which is assumed to be essential for the activity. In addition, another hydrogen bond was observed with Asn30 (2.72 Å), whereas, in the case of lisinopril, binding interactions with 6VW1 (binding score = −4.7, RMSD = 1.3) are given in Figure 3C, and two hydrogen bonds also were recorded, one of them with Asn90 (3.50 Å), which is assumed to be essential for the activity. Furthermore, another hydrogen bond was observed with Asn30 (2.92 Å).

Finally, some ACEIs such as trandolapril, fosinopril, and moexipril have excellent binding scores (−5.60, −5.04, and −5.10, respectively), better than the native ligand NAG (−4.4), but, unfortunately, their binding modes are different. For trandolapril, two hydrogen bonds were observed with Asp30 and the third one with Gln95 (2.75, 2.75, and 2.91 Å). For fosinopril, one hydrogen bond was observed with Gln96 (4.36 Å). For moexipril, three hydrogen bonds were observed with Asp30 (4.25, 3.16, and 3.36 Å).

### Molecular Dynamics Simulation

Considering it as an efficacious approach for validating the stability of the predicted docked ligand-*h*ACE2 complex, an all-atom molecular dynamics (MD) simulation study was performed. Adopting such a study would also provide valuable information regarding the dynamic behavior of both the ligand and *h*ACE2 protein as well as evaluate the ligand's key binding interactions with important catalytic site residues (Karplus and Petsko, 1990). Therefore, the predicted ligand–protein complexes, for both alacepril and lisinopril, as well as the glycosylated *h*ACE2 protein were enrolled within 100 ns all-atom MD simulation.

#### Trajectory Analysis of Ligand-hACE2 Complexes

The stability profile of both alacepril and lisinopril in complex with the human angiotensin-converting enzyme 2 (*h*ACE2) was monitored using the GROMACS command line *gmx_rmsd* to estimate their respective RMSD values throughout the simulation runs. Generally, RMSD provides an inference regarding the deviation extent for a group of atoms (protein, ligand, or even ligand–protein complex) to the respective initial reference structure (Schreiner et al., 2012). Thus, high RMSD values would be correlated to significant instability, being related to changes within the conformation of the investigated molecule. Moreover, ligands depicting high RMSD values, for their respective ligand–protein complex, would suggest inadequate ligand accommodation within the studied pocket across the adopted MD simulation time-frames (Liu et al., 2017).

**Figure 4:**
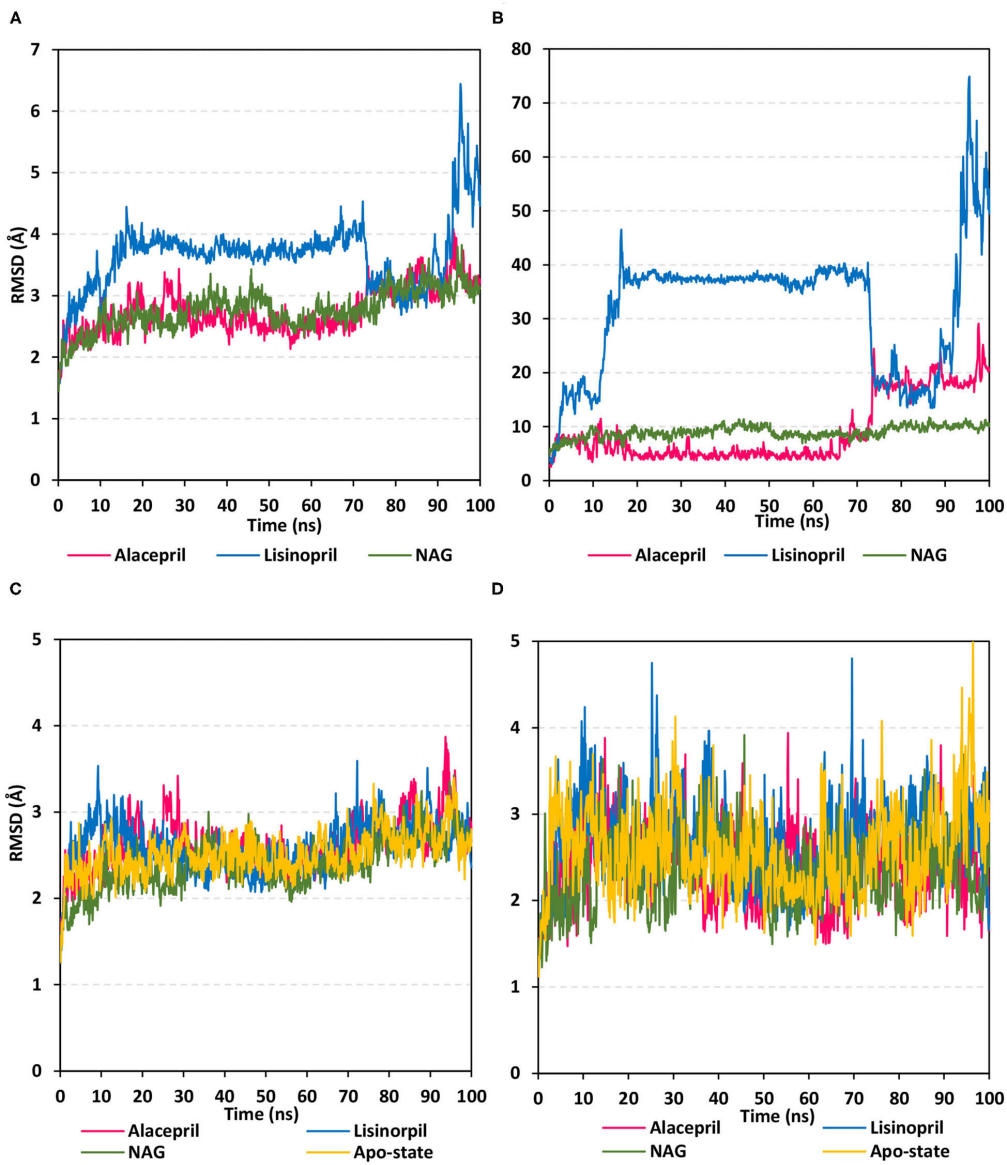
Analysis of RMSD trajectories for the ligand-*h*ACE2 protein complexes throughout 100 ns all-atom MD simulation. **(A)** Complex RMSD; **(B)** ligand RMSD; **(C)** protein RMSD; **(D)** binding pocket residues RMSD, relative to backbone vs. MD simulation time in nanoseconds. Alacepril/*h*ACE2 and lisinopril/*h*ACE2 complexes as well as glycosylated (NAG)-bound and apo-state (all glycans being removed) *h*ACE2 proteins are illustrated in pink, blue, green, and yellow colors, respectively.

Within the presented MD simulation, both investigated ligand–protein targets exhibited successful conversion following 20 ns of MD simulation start (Figure 4A). The obtained complex RMSD trajectories, in respect of their backbone, rises throughout the initial frames till the RMSDs level off at around 20 ns where the following trajectories proceeded around respective average values till the 70 ns of the MD simulation. It worth noting that the average RMSD values, throughout the plateau MD simulation interval (20–70 ns), were higher for lisinopril compared to alacepril (2.610 ± 0.20 Å vs. 3.786 ± 0.13 Å). The latter differential dynamic behavior confers a more stabilized and confinement accommodation for alacepril within the *h*ACE2 binding site throughout the plateau interval. However, both ligands converge around comparable RMSD values (~3.400 Å) where only the alacepril–protein trajectories were depicted steady till the end of the MD simulation at 100 ns. A second RMSD trajectory increase at the last 10 ns of the MD simulation was shown for lisinopril–protein complex tones, which further confirms a significant ligand shift out of the *h*ACE2 pocket. On the other hand, alacepril depicted a minimal increase within RMSD trajectories (from 2.316 to 3.110 Å) following the 70 ns suggesting a limited chance of the alacepril orientation within the *h*ACE2 pocket rather than a dramatic escape out of the binding site. All latter findings confer maintained binding of alacepril within the *h*ACE2 binding site. Compared to lisinopril, the alacepril–protein complex depicted comparable RMSD tones to those of NAG-bound (glycosylated) protein along the 100 ns all-atom MD simulation run. All above findings suggest a more preferential binding for alacepril, over lisinopril, within the *h*ACE2 NAG-binding site.

Further investigation of ligand stability within the protein binding site was proceeded through monitoring the ligand RMSD tones (Figure 4B). Monitoring these trajectories would provide valuable information regarding the conformational/orientation of the simulated ligands in respective to their binding pocket. Following convergence, the bound NAG molecule showed the steadiest RMSD tones (8.970 ± 1.14 Å) across the entire 100 ns all-atom MD simulation. Nevertheless, alacepril depicted the lowest RMSD trajectories (4.962 ± 1.28 Å) around the 20–70 ns MD simulation run being at ~1.5 Å RMSD values below those of its respective ligand–protein complex. With only limited fluctuations, the alacepril RMSD tones emphasize its preferential accommodation of the *h*ACE2 NAG-binding site as compared with lisinopril. The latter ligand depicted an extreme orientation/conformation shift relative to its initial coordinates (37.542 ± 0.92 Å) following 20 ns and up to 70 ns.

Beyond the 70 ns MD simulation runs, both ACEIs ligands exhibited comparable trajectories around 75–90 ns with the highest fluctuations being assigned for lisinopril. Finally, another elevated lisinopril RMSD values (> 50 Å), near the end of the MD simulation timeframe, suggested that lisinopril has left the protein interaction side while being strayed at the solvent site. Further monitoring of the pocket residue RMSD trajectories, with the crystal structure, was informative regarding the differential ligand binding within the *h*ACE2 NAG-binding site (Figure 4C). As expected, the highest RMSD tones (2.777 ± 0.48 Å) were assigned to lisinopril-pocket residues with high fluctuations being depicted around 25 ns and 70 ns (4.750 Å and 4.800 Å, respectively). Notably, pocket residues showed lower RMSDs with both alacepril and NAG binding (2.394 ± 0.42 Å and 2.346 ± 0.41 Å, respectively), as compared to *h*ACE2 with all glycans being removed (apo-state; 2.570 ± 0.49 Å), particularly near the end of the MD simulation. The latter behaviors confer preferential ligand-pocket mutual stability relationship for alacepril and NAG across the MD simulation runs.

For excluding the presence of any artifacts within the adopted MD simulation runs, the *h*ACE2 protein RMSD trajectories were monitored both for the apo (unbounded) and glycosylated (NAG-bound) states as well as in complex with both investigated ligands, alacepril, and lisinopril. Interestingly, the RMSD tones were comparable for the apo and complexed proteins since limited differential RMSD values were obtained across the 100 ns MD simulation window (Figure 4D). A little elevation of the protein RMSD tones, concerning their C-alpha atoms, was depicted at first frames of MD simulation and then an equilibrium plateau was achieved around an average RMSD of 2.558, 2.524, 2.661, and 2.611 Å, for apo, NAG, alacepril, and lisinopril-bound proteins, respectively. Such protein behavior is typical for optimum MD runs since all the applied constraints, before the simulation, were released and the protein starts to relax till reaching an equilibration state around which the RMSD revolves until reaching the MD simulation end. Showing comparable average RMSD values for apo *h*ACE2, relative to those for NAG, alacepril, and lisinopril-bound proteins could exclude the presence of differential significant secondary structure rearrangement/folding within the three MD simulation runs. The latter findings further correlate the RMSD complex trajectory fluctuations to the ligand behaviors rather than that of respective proteins within the MD simulation runs. It worth noting that all protein RMSDs reached comparable values (~2.600 Å) at the end of the MD run which further validate the 100 ns MD simulation time frame being able to bring both the apo, glycosylated and complexed proteins at comparable equilibration/relaxed states. Moreover, the latter dynamic behaviors further ensure sufficient conditioning stages before the production of the MD simulation runs.

**Figure 5:**
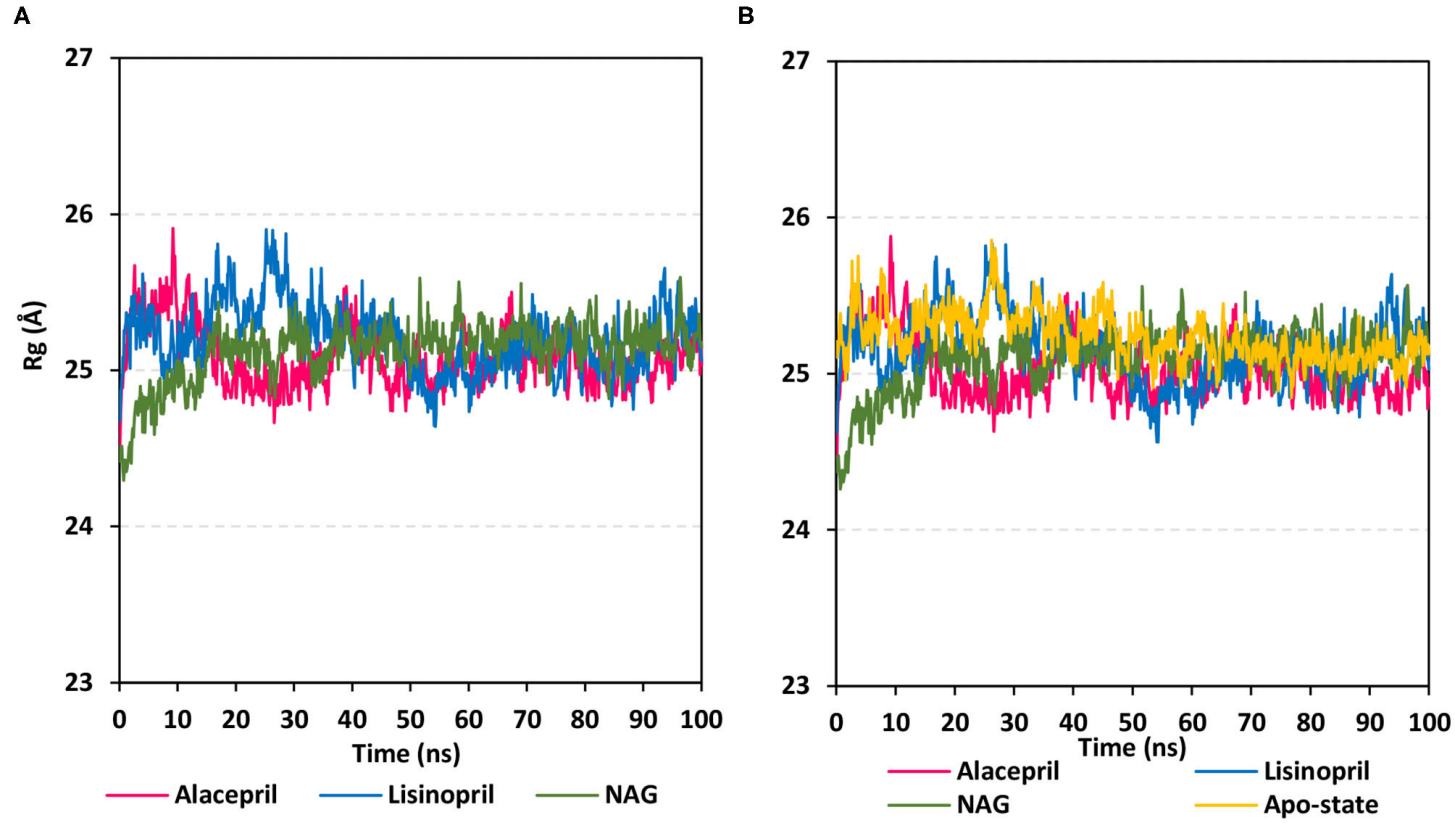
Global stability analysis of ligand-*h*ACE2 protein complexes throughout 100 ns all-atom MD simulation. **(A)** Complex Rg; **(B)** protein Rg, vs. MD simulation time in nanoseconds. Alacepril/*h*ACE2 and lisinopril/*h*ACE2 complexes as well as glycosylated (NAG)-bound and apo-state (all glycans being removed) *h*ACE2 proteins are illustrated in pink, blue, green, and yellow colors, respectively.

To gain more insight regarding the investigated complex stability, the radii of gyration (Rg) were monitored across the whole MD trajectories using the GROMACS “*gmx_gyrate*” command script. This stability parameter accounts for global stability of either ligand or protein ternary structure, where Rg is the mass-weighted RMSD for a group of atoms relative to their common mass center (Likić et al., 2005). Therefore, sustained stability/compactness of the investigated molecule would be inferred through depicted low Rg values achieving a plateau around an average value. Within the furnished study, the obtained Rg tones confirm the preferential stability of the alacepril-*h*ACE2 complex as compared to those of lisinopril (Figure 5). Steadier Rg trajectories were obtained for the alacepril complex with lower maximum, average, and minimum values (Table 2), suggesting compactness and stability of the ligand within the protein active site. Comparable values were depicted for alacepril and glycosylated (NAG)-bound protein complexes. The latter complex Rg findings were highly correlated with those of respective proteins. Minimal fluctuations and low Rg standard deviations were observed with alacepril and NAG as compared to that of lisinopril (25.03 ± 0.17 Å and 25.08 ± 0.19 Å; vs. 25.20 ± 0.21 Å, respectively). Interestingly, lower Rgs was assigned for the alacepril-bound and glycosylated (NAG) *h*ACE2 proteins with the protein's apo-state (25.23 ± 0.15 Å) suggesting a more compacted secondary structure upon ligand binding as well as protein glycosylation. All obtained Rg findings showed high agreement with the previous RMSD analysis confirming preferential better stability of alacepril over lisinopril within the *h*ACE2 NAG-binding site.

**Table 2:** The Rg values for investigated ligand-*h*ACE2 complexes across the all-atom MD simulation.

	**Alacepril-*****h*****ACE2 complex**			**Lisinopril-*****h*****ACE2 complex**			**Glycosylated (NAG)** ***h*****ACE2**		
**Reference atom group**	**Maximum (Å)**	**Average (Å)**	**Minimum (Å)**	**Maximum (Å)**	**Average (Å)**	**Minimum (Å)**	**Maximum (Å)**	**Average (Å)**	**Minimum (Å)**
Complex	25.78	25.08 ± 0.09	24.49	25.90	25.20 ± 0.21	24.63	25.59	25.12 ± 0.19	24.29
Protein	25.75	25.03 ± 0.17	24.45	25.88	25.15 ± 0.21	24.56	25.57	25.08 ± 0.19	24.26

#### Protein Flexibility and Root-Mean-Square Fluctuation of Target Residues

For gaining more insights regarding the stability of the complex binding site, the per residue rence root-mean-square fluctuation (ΔRMSF) profile was estimated for each ligand-bound protein relative to the *h*ACE2 apo-state. The individual backbone RMSF of each protein was estimated using the GROMACS “*gmx rmsf”* command line. This flexibility validation criterion provides information regarding the contribution of protein individual residues within the ligand/protein complex structural fluctuations. RMSF estimates the time evolution of the average deviation for each residue from its reference position within the minimized starting structures (Benson and Daggett, 2013). Adopting a ΔRMSF cut-off value of 0.30 Å was relevant for estimating the significant change within structural movements, where residues with > 0.30 ΔRMSF values were considered of decreased mobility (de Souza et al., 2019).

**Figure 6:**
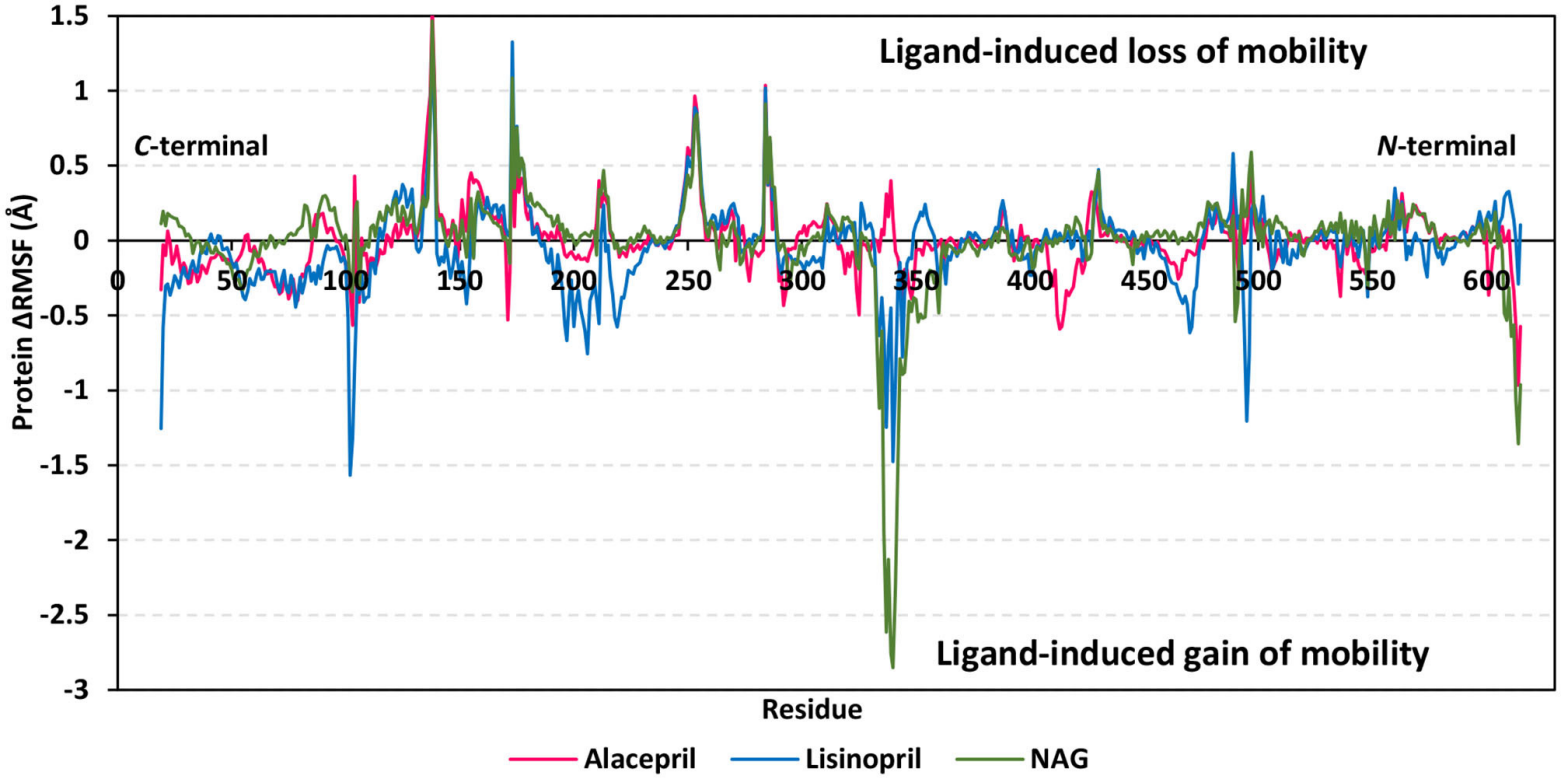
Relative ΔRMSF analysis of ligand-*h*ACE2 protein complexes throughout 100 ns all-atom MD simulation. Protein backbone ΔRMSF trajectories were determined from the independent MD-simulated *h*ACE2 apo-state against the complexed protein with alacepril, lisinopril, or NAG, which were shown as a function of residue number 19-to-619. Alacepril/*h*ACE2, lisinopril/*h*ACE2, and glycosylated (NAG)/*h*ACE2 complexes are illustrated in red, blue, and green colors, respectively.

Findings within Figure 6 showed expected terminal-free residue behavior with high negative ΔRMSF values since they are most likely to fluctuate at the highest deviations in comparison to core residues the thing that is typically depicted in well-behaved MD simulation. However, a different terminal-free residue pattern was assigned for each ligand. Lower RMSF negative values or even positive RMSF values were depicted for alacepril and NAG, respectively, for the *C*-terminal-free residues and vicinal residues. Since the *h*ACE2-NAG pocket residues are at proximity to the protein *C*-terminal side, such findings confer more stabilized alacepril and NAG-protein complexes as compared to lisinopril. At the *N*-terminal, lower negative RMSF values were assigned to lisinopril relative to alacepril and NAG, suggesting that *N*-terminal-free residues and vicinal residues might impact lisinopril-protein binding through MD simulation. As these latter residues are at > 30 Å distant from the reference *h*ACE2-NAG binding site, they may be highly correlated to stabilization of lisinopril following the dramatic conformational/orientation shift beyond 20 ns and up to 70 ns of the MD simulation run.

Concerning core protein residues, the three bounded ligands induced significant limited mobility (ΔRMSF > 0.3 Å) for *h*ACE2 residues at four distinct residue ranges including; range-I (134–140), range-II (173–178), range-III (248–256), and range-IV (284–286). The earlier two residue ranges-I and -II exhibited the greatest immobility with ΔRMSF values up to 1.55 Å and 0.91 Å, respectively. on the other hand, the other two less mobile residue ranges (-III and -IV) were at comparable ΔRMSF trajectories across the designated MD simulation window. Within the four top immobile residue ranges, the ΔRMSF trajectories for the three bound ligands were depicted as comparable. It worth noting that residues within the four residue ranges are at distances being > 29 Å from the bounded ligands the thing that can infer the impact of ligand binding site to induce stabilization of the protein secondary structures distant from the NAG-binding site.

Regarding residues with the highest fluctuations, there is a general trend of high negative RMSF values being assigned to the lisinopril-bound protein residues. Designated residue ranges (101–110, 195–220, and 462–473) exhibited high negative ΔRMSF values in particular for the protein in complex with lisinopril. Nevertheless, residues at these latter ranges showed limited flexibility regarding both alacepril and NAG-bound protein. Notably, one residue range (333–359) did not exhibit a similar pattern to the above highly mobile or immobile ranges, where residues of both lisinopril and NAG-bound protein were of great fluctuation/flexibility (maximum ΔRMSF −1.48 and −2.85 Å, respectively). On the contrary, positive ΔRMSF values (up to 0.40 Å) were assigned for the latter contradictory residue range up on alacepril binding suggesting the great impact of these residues on the alacepril-protein binding, which may be highly related to the suggested second conformation/orientation of alacepril following the 70 ns MD simulation run.

Further comparative analysis of the furnished ΔRMSF trajectories for the key residues lining the *h*ACE2-NAG binding site permitted more insights regarding differential ligand-protein interactions. To the most interest, several pocket residues illustrated significant immobility with a ΔRMSF value of > 0.30 Å for alacepril-bound protein (Table 3). Pocket residues including Asn90, Leu91, Leu560, and Ser563 depicted the highest ΔRMSF values being the most positive for Leu91 suggesting the residue's key role in alacepril-pocket anchoring. Concerning the pocket residues of the NAG-bound protein, Asn90 and its vicinal residues (Leu91 and Thr92) depicted significant rigidity. This was not surprising since crystallized NAG molecule is linked to *h*ACE2 at Asn90 within *h*ACE2 crystal structure. This observation ensures the stability of NAG as well as alacepril within the binding site along with the MD simulation frames. Moreover, the ability of alacepril to exhibit comparable immobility pattern or Asn90 and vicinal residues further emphasize the competitive capability of alacepril to replace NAG at its binding site. Moving toward the protein in complex with lisinopril, only Leu560, and Ser563 showed relevant rigidity with ΔRMSF values at the borderline (0.250 and 0.258 Å, respectively) being lower than those depicted with alacepril. It worth mentioning that several lisinopril-pocket residues, even those at the initial docking study, exhibited significant flexibility/fluctuations with ΔRMSF being of negative values (−0.035 to −0.264 Å). This finding can be correlated with the earlier suggestion that lisinopril has left the *h*ACE2-NAG binding site exhibiting dramatic orientation/conformation shift. All above ΔRMSF analysis infer the inferior impact of lisinopril, as compared to alacepril and NAG, on the immobility/stability of the protein pocket residues. Therefore, the ΔRMSF analysis is considered relevant as it came in great agreement with the above ΔRMSD and Rg findings suggesting the higher alacepril-*h*ACE2 complex stability relative to that of lisinopril.

**Table 3:** Calculated ΔRMSF^a^ trajectories of ligand-*h*ACE2 proteins along with the MD simulation.

**Residues of *h*ACE2-NAG binding site**	**Alacepril**	**Lisinopril**	**NAG**
Ala25	−0.114	−0.236	0.148
**Lys26**	−0.037	−0.264	0.146
Asp30	0.239	−0.182	0.030
**Lys31**	0.286	−0.252	0.043
**Asn90**	0.381	−0.146	0.295
**Leu91**	0.404	−0.076	0.300
**Thr92**	0.053	−0.035	0.274
Val93	0.082	−0.06	0.204
Leu95	0.028	−0.053	0.221
Gln96	−0.006	−0.039	0.130
Ala387	0.091	0.198	0.070
Gln388	0.217	0.267	0.089
Pro389	0.199	0.191	0.054
**Leu560**	0.315	0.250	0.005
**Ser563**	0.314	0.258	0.241
Glu564	0.197	0.105	0.088

a *Relative difference root-mean-square fluctuation (ΔRMSF) was estimated for each ligand-bound protein relative to hACE2 apo-state being without any glycan. Residues exhibiting significant immobility (ΔRMSF above 0.30 Å) are only written in bold and representative ΔRMSF value is highlighted*.

#### Conformational Analysis Across Selected Trajectories

**Figure 7:**
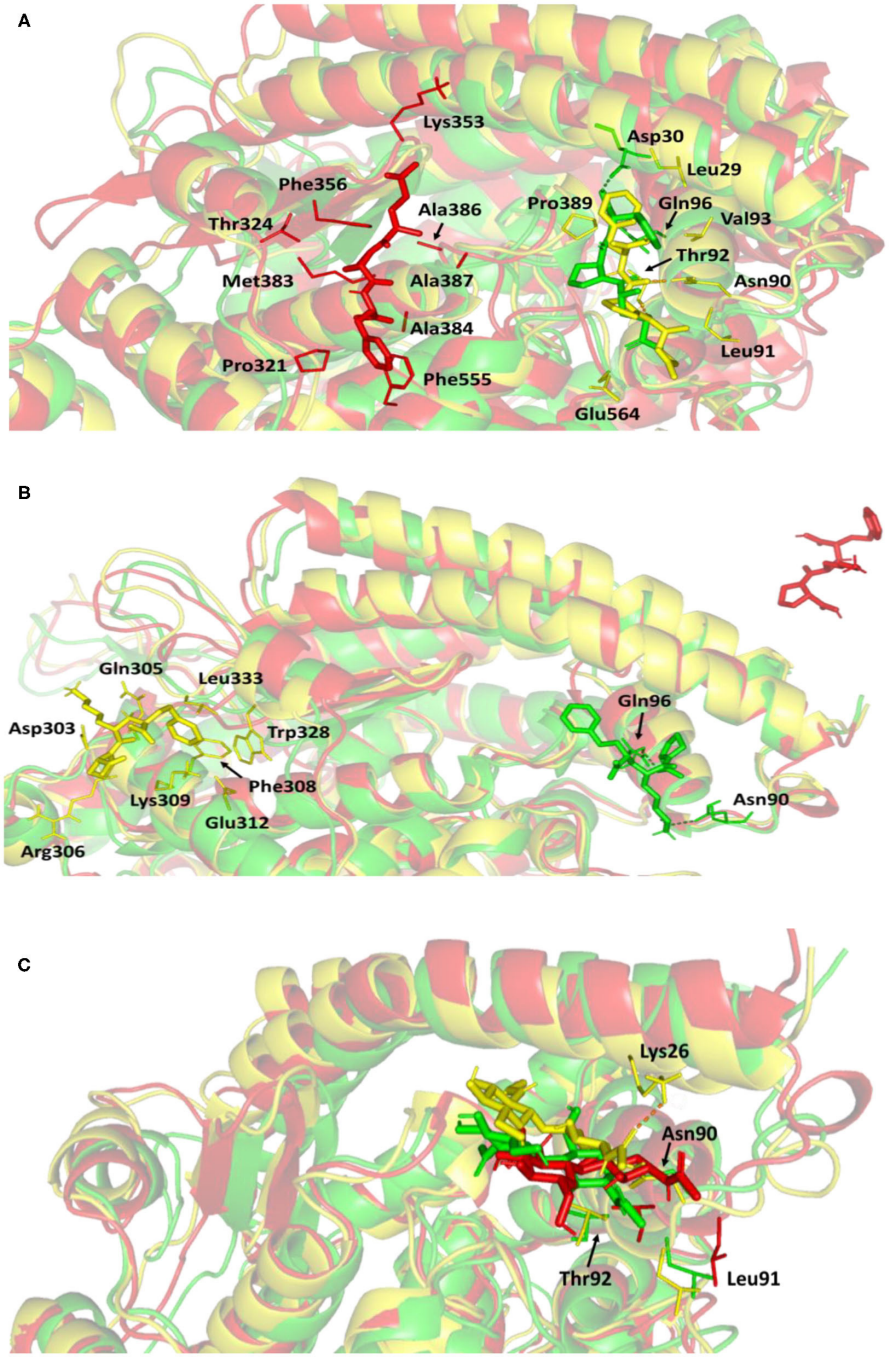
Conformations of the ligand-protein complex at *h*ACE2 binding site through selected trajectories. **(A)** Alacepril; **(B)** lisinopril; **(C)** NAG. Protein is represented in green, yellow, and red cartoon 3D-representation corresponding to initial (0 ns), dynamic equilibrium (70 ns), and last (100 ns) extracted trajectories, respectively. The key binding residues (lines), ligands (sticks), and hydrophilic interactions (hydrogen bonding; dashed lines) are all presented in colors corresponding to their respective extracted trajectory.

For gaining more insight regarding the newly adopted ligand–protein conformations by each ligand within the late MD simulation runs, the selected frames of each system were extracted and minimized to a gradient of 0.001 Kcal/mol/A^2^ using MOE software for further analysis of key changes. Figure 7A illustrates the comparative conformations of the alacepril-protein complex at 0, 70, and 100 ns. Interestingly, there is no significant orientation change for the ligand within the *h*ACE2 binding site between the time frames 0 and 65 ns. There was only a relevant shift toward the main chain of the Asp90 residue furnishing significant hydrogen bonding with its backbone amide. Such a shift caused a loss of the initial hydrogen bond with Asp30 and Gln96. Stabilization of alacepril within its new conformation/orientation was further mediated by several non-polar residues, including Leu29, Lue91, Val93, Pro389, in addition to the Cβ of Glu564 side chain.

Concerning the ligand conformation at frame 100 ns, a more significant shift was depicted by alacepril toward a transient opened cleft at proximity to the SARS-CoV-2 spike-protein recognition domain-III. Such shift came in good agreement with the RMSD fluctuation following the 70 ns. Notably, the ligand was mainly maintained within this transit cleft through hydrophobic interaction with pocket lining residues. Being anchored at proximity to the protein's hydrophobic residues, Pro321, Phe356, Ala383, Ala386, Ala387, and Phe555, favored non-polar interactions were depicted with the ligand's terminal phenyl ring and pyrrolidine hydrophobic cage.

Validating the stability of alacepril within this transit cleft was achieved through extending the MD simulation. The last alacepril frame at 100 ns was extracted, minimized, and then proceeded within an extra 50 ns all-atom MD simulation adopting the same parameters at the initial 100 ns MD simulation run. Notably, alacepril showed great stability across the additional trajectories where the RMSD tones for the alacepril-*h*ACE2 complex and protein were maintained at low values (2.511 ± 0.33 Å and 2.482 ± 0.34 Å, respectively), following convergence (Supplementary Figure 1). Showing minimal fluctuations across the extended trajectories confirms the stability of alacepril at the transit cleft being still bounded with the pocket residues.

Concerning the lisinopril-*h*ACE2 complex, a more dramatic conformational and orientation shift was depicted for the ligand (Figure 7B). Throughout the dynamic equilibration shown from 20 to 70 ns, lisinopril was anchored at a distant pocket seated at ~25.00 Å from the initial *h*ACE2 binding site. These deviations can be correlated to the high complex RMSD-Cα fluctuations (Figure 4A) and the high maximum value of complex Rg (25.90 Å) compared to the alacepril–protein complex system. At this new distant pocket, relevant hydrophobic contacts between lisinopril and lining residues (Phe308, Trp328, and Leu 333) greatly mediated the ligand-protein complex stability. Interestingly, this distant pocket is near the *N*-terminal free residues and their vicinal residues. The binding of lisinopril within this distant pocket can explain the lower negative ΔRMSF trajectories of the *N*-terminal free residues, as compared to alacepril and NAG. Therefore, it is suggested that these residues impose a crucial role in stabilizing the lisinopril-protein complex within the 20–70 ns timeframe. Based on the furnished results, inferior stability within the *h*ACE2 binding site was assigned to lisinopril as compared to alacepril. The latter was further confirmed since lisinopril was found at the solvent side as being drifted away from the *h*ACE2 protein at the end of the MD simulation (100 ns).

Investigating the conformational changes for the glycosylated *h*ACE2 protein showed that NAG was retained within the binding pocket along with the whole MD simulation timeframe (Figure 7C). There is a quite comparable orientation for the NAG conformation at the 70 ns frame concerning its initial position at 0 ns time. Polar hydrogen bonding with the pocket hydrophilic residue, Lys26, was shown to provide extra stability for the NAG at the binding site. On the other hand, significant movement of NAG, as well as the pocket residues (Asn90, Leu91, and Thr92), was illustrated at the end of the MD simulation. Despite that, these particular pocket residues have exhibited relevant immobility with high positive ΔRMSF values (Table 3), a significant change in their respective position as depicted. This could raise the assumption that NAG is not fully occluding the binding site of interest the thing that could make it at least partially accessible across the designated MD simulation. Proving such a concept would provide relevant evidence that small druggable molecules, like alacepril, could manage to accommodate the *h*ACE2-NAG binding site of the glycosylated protein.

#### Extent of hACE2 Binding Site Coverage by NAG

To speculate the possibility of small ligand inhibitors to accommodate the *h*ACE2-NAG binding site, an investigation of the extent of *h*ACE2 binding site coverage by NAG was within the glycosylated protein was proceeded. The GROMACS “*gmx sasa*” tool was used to compare the solvent-accessible-surface area (SASA) of the binding region in the absence and presence of glycan. Generally, SASA correlates for the molecular surface area being assessable to solvent molecules providing a quantitative measurement about the extent of protein/solvent interaction (Pirolli et al., 2014). The analysis was calculated for the atoms of lining residues comprising the *h*ACE2 binding site using spherical probes estimating the area exposed to the solvent. The% area of the binding site coverage was calculated as the percentage difference between the solvent-exposed area in the presence and absence of NAG. The solvent-sized probes (small radii, 1.4 Å) were applied to detect the binding site regions being within direct contact with the glycan. These small-sized probes are appropriate for checking the accessibility of small drug-like molecules. However, larger probes (5–10 Å radius) are more correlated with more accurate SASA calculations for macromolecules including antibodies and protein-based molecules (Urbanowicz et al., 2019). Three different probe sizes (1.4 Å, 7.2 Å, and 10 Å radii) were utilized for investigating distinct types of binding site coverage (Grant et al., 2020).

**Figure 8:**
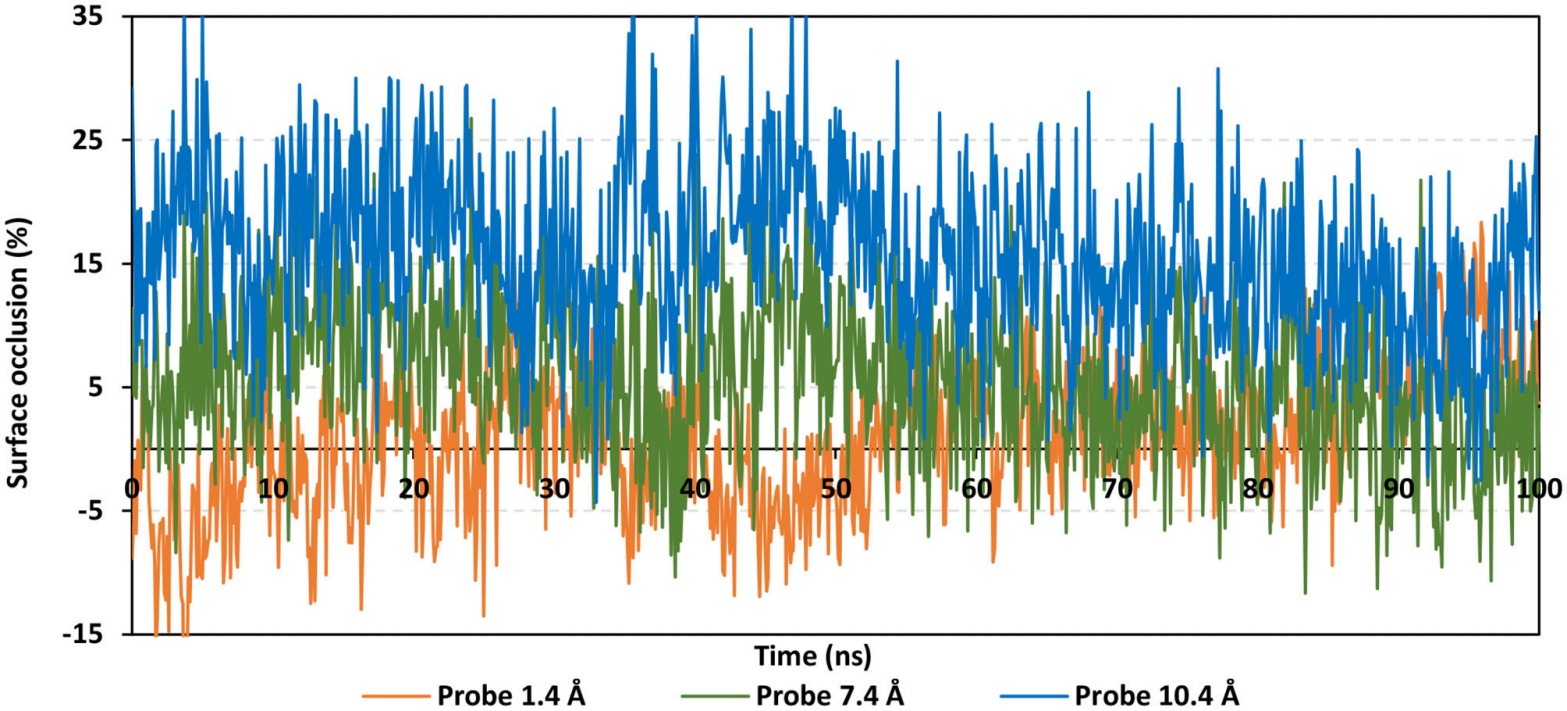
Extent of *h*ACE2 binding site coverage via SASA analysis along with the time evolution 100 ns all-atom MD simulation. Surface occlusion is defined as the surface percentage being covered via NAG being calculated relying on the SASA differences for the binding site surface in the presence and absence of NAG glycans. Three different probe sizes (1.4, 7.2, and 10 Å) were utilized for calculating the SASA values. Data are represented as % surface occlusion vs. the MD simulation time in nanoseconds.

Findings of the adapted SASA calculations illustrated insignificant binding site coverage by NAG (1.318 ± 5.79%) using the small probes (1.4 Å) (Figure 8). On the other hand, moderate% surface occlusion was depicted on larger probes, 7.2 Å and 10.4 Å, where less than half of the binding site was covered by NAG (5.502 ± 6.40% and 15.874 ± 6.86%, respectively) throughout the MD simulation run. With several SASA trajectories having negative% area coverage values, the simulated NAG molecule is considered to have a lower number of interactions with the binding site residues as well as non-complete coverage particularly with the 1.4 Å sized probes. Based on the above SASA findings, the binding site of interest has shown significant accessibility for small drug-like molecules as compared to peptidomimetic and antibody-related macromolecules during the simulation. Evaluation of the binding interactions for alacepril within the significant accessible *h*ACE2-NAG binding site would identify the “hot spot” residues showing long-term hydrophilic interaction-related stabilization of the ligand within the binding site. Such information is highly relevant for understanding the evolution of ligand stability inside the protein pocket.

#### Binding Interaction Analysis

Investigating the hydrogen bond network interactions between the *h*ACE2 residues and alacepril, over the 100 ns MD simulation, was considered crucial for understanding the observed conformational changes and stability of ligand–protein complexes. Using the VMD “Hydrogen bonds” tool, it was useful to explore the established ligand-protein hydrogen bond interactions and their relative frequencies (Humphrey et al., 1996). The cut-off values for hydrogen bond (Donor-H…Acceptor) distance and angle were assigned at 3.0 Å and 20°, respectively (de Souza et al., 2019; Albuquerque et al., 2020).

**Figure 9:**
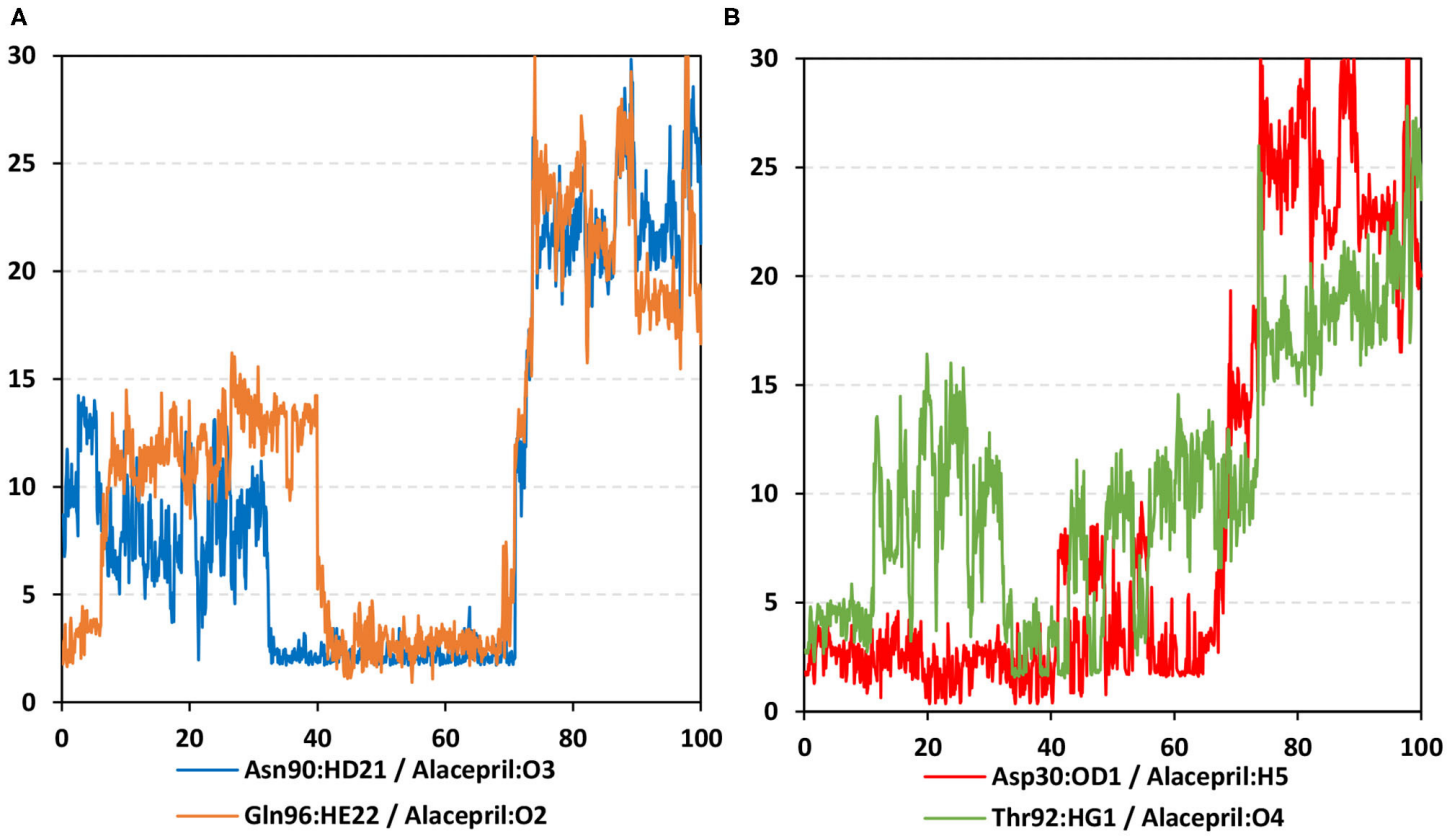
Time-evolution of hydrogen bond distances for alacepril with *h*ACE2 key binding residues vs. 100-ns MD simulation time. **(A)** Asn90 and Gln96; **(B)** Asp30 and Thr92. The Y- and X-axes correlate to the apparent hydrogen bond (Donor-H…. Acceptor) distance in Å and MD simulation time in nanoseconds, respectively.

As expected, the hydrogen bond pairs between alacepril and either Asn90 or Gln96 were of the highest frequency, 55 and 37%, respectively, mediating the ligand–protein stabilization within the MD simulation interval 30–70 ns (Figure 9A). Following the 70 ns MD simulation frame, the latter polar interactions were lost as alacepril adopted the new shifted orientation/conformation at the transient opened cleft near the SARS-CoV-2 spike-protein recognition domain-III. On the other hand, the initial hydrogen bond pair Thr92:HG1-Alacepril: O4 was lost following the 10 ns of the MD simulation starts showing a minimal frequency of 4% (Figure 9B). This confers a limited contribution of Thr92 for the stabilization of the alacepril-*h*ACE2 complex.

Surprisingly, the initial hydrogen bond interaction between alacepril and Asp30 was conserved up to 40 ns of the MS simulation. Despite limited fluctuations up to 8 Å hydrogen bond distances, the Asp30:OD1-Alacepril:H5 hydrogen bond pair was quite relevant particularly between the 57 ns and 65 ns MD simulation frames. Typically, Asp30 is reported as a key polar residue for anchoring the SARS-CoV-2 spike glycoprotein on the receptor-binding domain of hACE2 through hydrogen bond interaction with Lys417 of the spike protein (Shang et al., 2020; Wang et al., 2020). Therefore, the depicted occurrence of hydrogen bonding between alacepril and Asp30 for more than 40 ns arose the promising role of alacepril to counter SARS-CoV-2/host entrance. It is suggested that polar anchoring of alacepril with any of the polar residues, involved at the S-protein-ACE2 connective interface, would probably impact both subdomains binding affinity (Hoffmann et al., 2020). Both suggested scenarios would halt the crucial stage of COVID-19 infection which is the virus-host membrane fusion and subsequent release of viral payload RNA into the host cytoplasm.

#### Binding-Free Energy Calculations

By illustrating the accessibility of the glycosylated site, we carried out an investigation of the differential binding affinity for the small molecules of interest and the *N*-glycan chain. Illustrating the potentiality of alacepril to compete with *N*-glycan for engaging the cavity near the glycan site would be beneficial to suggest an ability for disrupting the glycosylation process of the *h*ACE2, leading to the modulation of *h*ACE2-RBD interactions. Based on this, the following binding-free energy calculation was adopted to understand the nature of the alacepril-protein binding, explore the comparative alacepril/*N*-glycan-binding site affinity, and obtain more information concerning alacepril/residue contribution (Cavasotto, 2020). The MD-based Molecular Mechanics/Poisson Boltzmann Surface Area (MM/PBSA) approach was adopted for the designated binding-free energy calculations, using the “*g_mmpbsa*” tool on GROMACS. The approach accounts for more accurate ligand-protein affinity as compared to the most sophisticated flexible molecular docking technique (Kumari et al., 2014). Generally, MM/PBSA estimates binding-free energy as a contribution of several energy terms through these given Equations (Kumari et al., 2014):

$$\begin{array}{l} \Delta G_{\text{binding}}=G_{\text{complex}}-\left(G_{\text{ligand}}+G_{\text{protein}}\right)\\ Gx=\left(E_{\text{MM}}\right)-TS+G_{\text{solvation}}\\ E_{\text{MM}}=E_{\text{bonded}}+\left(E_{\text{vdW}}+E_{\text{electrostatic}}\right)\\ G_{\text{solvation}}=G_{\text{polar}}+\gamma SASA+b \end{array}$$

where Δ*G*_binding_ is the binding-free energy correlating to ligand–protein binding where the higher negative energy values infer greater protein–ligand affinity. The energy terms *G*_*complex*_, *G*_protein_, and *G*_ligand_ are the total free energies of ligand–protein complex, isolated protein, and isolated ligand in the solvent, respectively. Vacuum MM potential energy (*E*_MM_) together with the entropic contribution to free energy (*TS*) and free energy of solvation (*G*_solvation_) provided the total free energy of protein, ligand, or ligand–protein complex (*E*_X_). Terms *T* and *S* denote temperature and entropy, respectively, while *E*_MM_ was calculated based on molecular mechanics force-field parameters. Using the solvent-accessible surface area (SASA)-Non-polar Model, the *G*_solvation_ energy term comprises polar and non-polar parts, where the latter was estimated via SASA and fitting constant (*b*). Finally, *G*_polar_ is to be solved from the Poisson-Boltzmann equation.

Typically, the binding-free energy should be estimated from the MD simulation trajectories depicting stabilized protein–ligand systems. Thus, the free energy calculation was adopted across the 30–70 ns and last 20 ns intervals where representative frames were extracted and saved to be enrolled within the calculation of each energy term. Adopting these specific time frames was rationalized by the above complex backbone RMSD analysis where equilibrated plateau tones were illustrated within the 30-to-70 ns and last 20 ns timeframe interval (Figure 4).

Interestingly, the Δ*G*_binding_ of the alacepril-*h*ACE2 complex was estimated at higher negative values around the 30–70 ns MD simulation interval as compared to that at 80–100 ns (−51.812 ± 17.494 kJ/mol vs. −37.898 ± 10.993 kJ/mol, respectively) (Table 4). A similar pattern was shown with lisinopril where its respective free-binding energy was lower across the last 20 ns MD simulation timeframes as compared to 30–70 ns ones. This less favored lisinopril–protein-binding energy came in good agreement with highly fluctuated RMSD and Rg tones near the end of the MD simulation. On the other hand, the Δ*G*_binding_ of the NAG-*h*ACE2 complex was of comparable values (−45.384 ± 47.279 and −48.729 ± 34.272 kJ/mol) across the two designated MD simulation time frames. The latter was expected since NAG depicted the steadiest complex RMSD trajectories along the whole MD simulation run.

**Table 4:** Binding-free energies calculations (± standard deviation; SD) for the investigated ligand-*h*ACE2 protein complexes.

**Energy terms (kJ/mol ± SD)**	**30–70 ns**			**80–100 ns**		
	**Alacepril**	**Lisinopril**	**NAG**	**Alacepril**	**Lisinopril**	**NAG**
Δ*G*_Van der Waal_	−101.954 ± 19.491	−121.265 ± 7.925	−3.784 ± 44.849	−76.329 ± 16.017	−30.148 ± 30.735	−7.143 ± 52.568
Δ*G*_Electrostatic_	−24.080 ± 21.066	−134.936 ± 45.623	−142.512 ± 26.408	−9.010 ± 12.093	−99.892 ± 188.971	−137.297 ± 28.240
Δ*G*_Solvation_ (Polar)	85.130 ± 25.313	231.848 ± 0.866	109.767 ± 18.873	59.004 ± 21.113	111.265 ± 11.102	104.107 ± 43.007
Δ*G*_Solvation_ (SASA)	−10.908 ± 1.736	−14.900 ± 0.929	−8.855 ± 2.190	−11.563 ± 2.503	−4.288 ± 4.118	−8.396 ± 2.045
Δ*G*_Binding_	−51.812 ± 17.494	−39.255 ± 49.430	−45.384 ± 47.279	−37.898 ± 10.993	−23.063 ± 21.715	−48.729 ± 34.272

Dissecting the furnished alacepril-*h*ACE2 Δ*G*_binding_ around both MD simulation intervals showed a preferential contribution of the hydrophobic van der Waal interactions as compared to that of the electrostatic energy term. However, the significant occupancy of the depicted hydrogen-bond interaction analysis around 30–70 ns MD simulation interval can suggest a somewhat balanced contribution between both energy terms. Moreover, the low electrostatic (Δ*G*_electrostatic_) contribution for 80–100 ns binding energy came in great agreement with the above bonding analysis findings where hydrogen bonding between alacepril and *h*ACE2 residues were limited as well as of minimal frequencies/occupancies. It worth mentioning that moderate Δ*G*_solvation_ energy term for alacepril at 30–70 ns interval (85.130 ± 25.313 kJ/mol) is considered favored for ligand–protein binding. as being balanced for the advent of the high electrostatic and Van der Waal energy contribution (−126.034 kJ/mol) afforded by alacepril scaffold. The latter compensated Δ*G*_solvation_ energy contribution further ensures the favored stability of the alacepril-*h*ACE2 complex since ligand binding is a solvent-substitution process.

For lisinopril, almost equal van de Waal/electrostatic energy contributions were assigned for the first MD simulation interval, whereas the electrostatic energy term depicted dominant free-binding energy contribution, nearly 3-fold higher than that of **Δ*G***_**Van der Waal**_, within the last 20 ns. This came in great agreement with the above conformational analysis since the ligand showed an escape from the pocket side while becoming more solvent-exposed near the end of the MS simulation run. It worth mentioning that much higher Δ*G*_Solvation_ values were depicted for the lisinopril-*h*ACE2 complex imposing a great penalty for the total free-binding energy calculation and ligand-protein binding. This could partially explain why lisinopril would exhibit dramatic conformational/orientation shift beyond 20 ns as well as moving toward the solvent side while escaping the protein interface at the end of the MD simulation run.

Considering the NAG, the van der Waal energy term contribution was insignificant within the ligand–protein free-binding energy calculation depicting very low negative values across both designated MD simulation intervals. On the contrarily, the electrostatic energy term was of higher contributions across both MD intervals. This differential Δ*G*_Van der Waal_/Δ*G*_Electrostatic_ pattern could be reasoned for the chemical nature of NAG scaffold being rich in polar oxygen-based functionalities, which serve as excellent hydrogen bond donor/acceptor. The latter is expected to impose a higher energy penalty upon close contact with the hydrophobic residues lining the *h*ACE2-pocket (Leu91, Val93, Ala387, and Leu560). Additionally, the polar sugar scaffold of NAG imposed high unfavored Δ*G*_solvation_, which negatively impacted the ligand–protein binding since such a process is a solvent-substitution approach.

**Figure 10:**
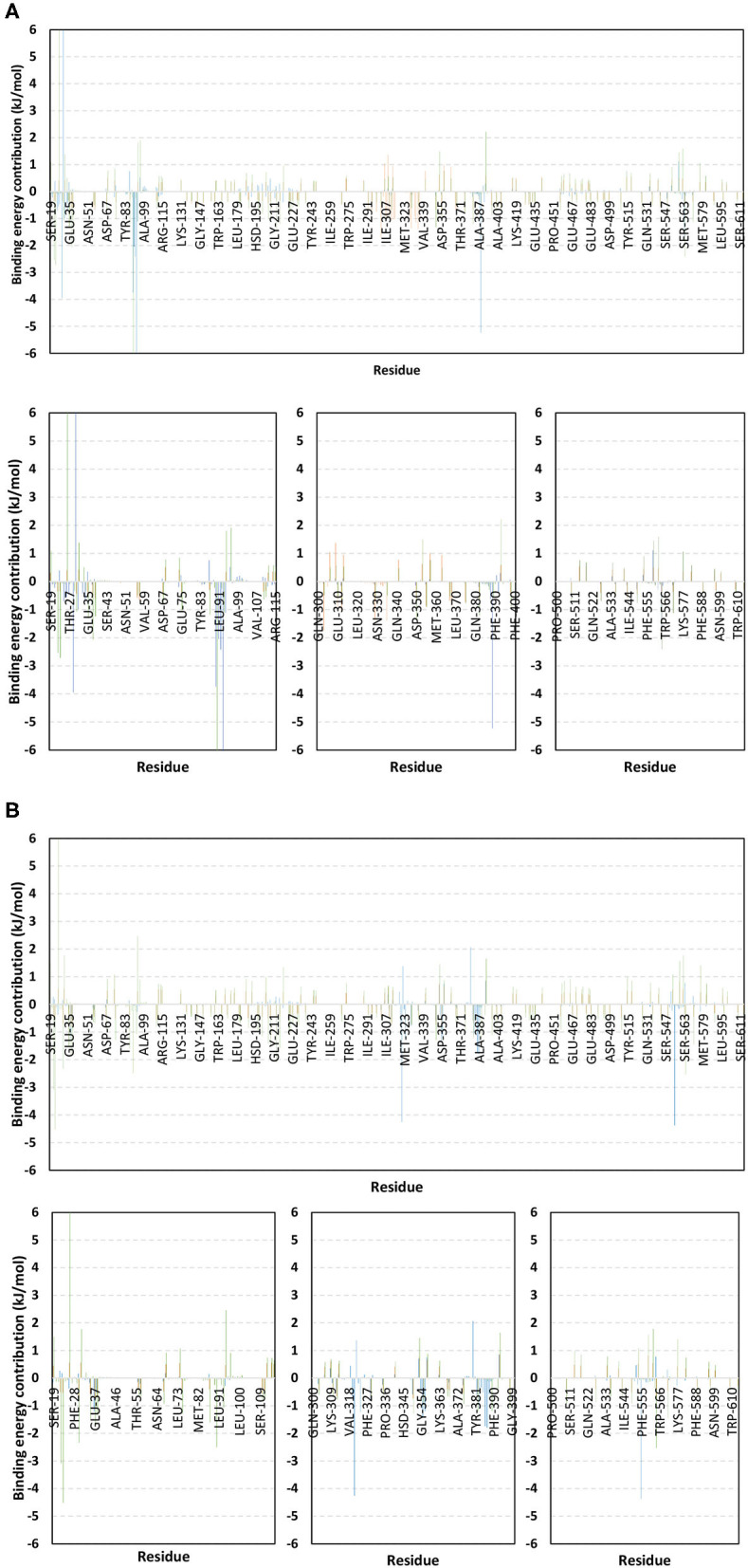
Binding-free energy/residue decomposition illustrating the protein residue contribution at alacepril-*h*ACE2 protein complex Δ*G*_binding_ calculation. The residue-wise energy contributions across **(A)** 30–70 ns and **(B)** 80–100 ns MD simulation timeframes were represented in blue, brown, and green colored bars for alacepril, lisinopril, and NAG, respectively. Lower panels are expanded versions of three designated residue regions (19–115, 300–400, and 500–614) of the upper panels.

For identifying the critical residues involved within the binding of ligands with *h*ACE2 protein, the residue-wise energy contribution to the obtained Δ*G*_binding_ was also estimated using *g_mmpbsa* (Figure 10) (Kumari et al., 2014). As a general observation, both alacepril and NAG depicted high residue-wise energy contribution near the *C*-terminal, particularly across the 30–70 ns MD simulation interval (Figure 10A). The latter is in great agreement with the previously discussed ΔRMSF analysis where the *C*-terminal free residues and their vicinal amino acids showed significant immobility with high positive values (ΔRMSF > 0.30 Å). This further confirms the significant stabilized binding of alacepril within the *h*ACE2-NAG pocket within this simulation interval. Comprehensive analysis of residue-wise energy contribution for alacepril across 30–70 ns showed significant contributions by Asn90 and Thr93, conferring their key role for stabilizing the alacepril-protein complex. Moreover, several key residues, which have participated in relevant within the initial docking analysis, showed significant contributions to the calculated Δ*G*_binding_. The high energy contribution by Asp30 came in great agreement with the previous hydrogen bonding analysis as the Asp30:OD1-Alacepril:H5 hydrogen bond pair was conserved for significant MD simulation frames. This high energy contribution further ensures the promising antiviral activity of alacepril in countering the SARS-CoV-2/host entrance through hampering the polar interaction role of Asp30 within *h*ACE2 spike-protein annealing and anchoring (Hoffmann et al., 2020). Other initial *h*ACE2 binding site residue and vicinal amino acids depicted significant contribution within alacepril-complex free-binding, including Leu29, Lue91, Val93, Pro389, and Glu312. These dominant non-polar energy contributions further confirm the superiority of Δ*G*_Van der Waal_ energy as compared Δ*G*_Velectrostatic_ energy term.

Moving toward the MD simulation 80–100 ns, residues of the transient opened cleft which is at proximity to the SARS-CoV-2 spike-protein recognition domain-III have depicted significant free-binding energy contributions (Figure 10B). The latter involves Glu37, Pro321, Asn322, Thr324, Asp355, Phe356, Gln380, Met383, Ala386, Ala387, and Phe555 residues. Notably, the highest energy contributions (−4.259 and −4.373 kJ/mol) were assigned for the aromatic hydrophobic residues (Pro321 and Phe555, respectively), while moderate contributions (−1.074 to −1.798 kJ/mol) were assigned to Phe356, Met383, Ala386, and Ala387 residues. The latter confers dominance of hydrophobic interactions (Δ*G*_Van der Waal_) for stabilizing alacepril at *h*ACE2 transient opened pit.

Moving toward the glycosylated *h*ACE2, a similar pattern of residue-wise energy contributions was depicted across the 30–70 ns and 80–100 ns intervals. Both pocket and vicinal residues showed significant contribution within the NAG Δ*G*_binding_ calculation. Across the 30–70 ns time frame, the highest energy contributions were assigned to Lys26 and Asn90 (6.184 and −6.867 kJ/mol, respectively), conferring their key role in NAG stabilization within the protein pocket. Other pocket/vicinal residues such as Glu22, Glu23, Asp30, Glu35, Glu37, Asp38, Glu87, Gln96, Asp213, Asp216, Arg393, Glu564, and Glu571, showed moderate energy contributions (−1.081 to −2.545 kJ/mol). Owing to the hydrophilic nature of these residues, an explanation of the dominant Δ*G*_electrostatic_ energy term contribution within the NAG free-binding energy calculation is to be rationalized. Regarding the last 20 ns interval, a general trend of increased residue-wise energy contributions was depicted for several residues, particularly those at proximity to *C*-terminal (Ser19, Glu22, Glu23, Lys26, Asp30, Glu35, Glu37, and Asp38). Nearly a 2-fold increase in the Lys26 energy contribution was depicted at 80–100 ns as compared to the 30–70 ns interval.

Other *N*-terminal pocket residues (Arg559, Lys562, Glu564, Glu571, and Lys577) showed a similar trend of increased residue-wise energy contributions. Contrarily, other pocket residues including Asp90, Val93, Gln96, and Arg393 showed lower energy contributions at the last 20 ns time with the highest descent for Asp90 being from −6.867 to −2.573 kJ/mol. Such differential pattern of residue-wise energy contribution shift came in great agreement with the previously described conformational analysis where a significant change in NAG respective position was depicted at the 100 ns frame. This further confirms the assumption that NAG is not fully occluding the binding site of interest the thing that could make it at least partially accessible across the designated MD simulation. Therefore, small druggable molecules, like alacepril, could manage to accommodate the *h*ACE2-NAG binding site of the glycosylated protein effectively.

Considering the last investigated complex, the lisinopril residue-wise energy contribution showed minimal values for the *C*-terminal free residues and their vicinal amino acids. This came in adherence with the above ΔRMSF analysis confirming the escape of lisinopril from the initial *h*ACE2-NAG binding site. Significant energy contribution was depicted for Asp299, Asp303, Arg306, Ile307, Phe308, Lys309, Glu310, Glu312, Lys313, Phe314, Phe315, Trp328, Glu329, Met332, Leu333, Asp335, and Pro336. In worth noting that these latter residues comprise the distant pocket, or its vicinal residues, being accommodated by lisinopril throughout the previously described conformational analysis along with the 30–70 ns interval. Interestingly, the balanced hydrophilic/hydrophobic nature of these residues could explain the comparable contributions of Δ*G*_electrostatic_ and Δ*G*_Van der Waal_ energy terms within the lisinopril-protein binding throughout 30–70 ns. As expected, a significant decrease within the latter residue-wise energy contribution profile was observed across the 80–100 ns trajectories, since lisinopril showed instability and dramatic shift toward the solvent side.

## Conclusion

A total of 14 ACEIs were subjected to virtual screening molecular docking against the spike protein of COVID-19. The tested drugs exhibited variable degrees of affinities toward the COVID-19 spike protein comparing to the native inhibitor. Alacepril and lisinopril were found to interact with COVID-19 spike protein by exhibiting the most acceptable rmsd_refine values and the best binding affinity through forming a strong hydrogen bond with Asn90, which is assumed to be essential for the activity, as well as significant extra interactions with other receptor-binding residues. Throughout the all-atom 100 ns MD simulation, alacepril depicted superior stability at the *h*ACE2 binding site for more than 70 ns, where the solvation energy was greatly compensated by the electrostatic and Van der Waal binding energies. SASA calculations for *h*ACE2 pocket in the presence and absence of glycan showed significant accessibility of the pocket for small drug-like molecules like alacepril. Moreover, alacepril mediated a stabilized favored hydrogen bond interaction with Asn30 which was conserved for significant MD simulation intervals. Depicting this favored hydrogen bond pair as well as the reported key role in *h*ACE2/SARS-CoV-2 spike-protein association introduces the promising action of alacepril to counter COVID-19/host entrance and subsequent release of viral payload RNA into the host cytoplasm through hampering *h*ACE2 spike-protein annealing and anchoring. Based on the furnished evidence, these drugs are recommended to be tested clinically for proposed activity against COVID-19. They may be tested either alone or in combinations. Also, our results may give a clear spot about SAR required for the spike-protein targeting drug to facilitate the future design and synthesis of new candidates against COVID-19.

## Data Availability Statement

The raw data supporting the conclusions of this article will be made available by the authors, without undue reservation.

## Author Contributions

AA-K: docking studies. SE and KD: molecular dynamic. IE: research plan. EE: data collection. AM: revision editing and references. MD: writing manuscript. All authors contributed to the article and approved the submitted version.

## Conflict of Interest

The authors declare that the research was conducted in the absence of any commercial or financial relationships that could be construed as a potential conflict of interest.
