# Supplementary material for: Molecular Docking and Dynamics Simulation Revealed the Potential Inhibitory Activity of ACEIs Against SARS-CoV-2 Targeting the hACE2 Receptor
Source: Front Chem. 2021 May 4;9:661230. doi: 10.3389/fchem.2021.661230 (PMC8129187; doi:10.3389/fchem.2021.661230)
Supplement: Supplementary file 1 [file Data_Sheet_1.docx]

**Molecular docking and dynamics simulation revealed the potential inhibitory activity of ACEIs against SARS-CoV-2 targeting *h*ACE2 receptor**

Ahmed A. Al‐Karmalawy^a^, Mohammed A. Dahab^b*^, Ahmed. M. Metwaly^c^, Sameh S. Elhady^d^, Eslam B. Elkaeed^e,f^, Ibrahim. H. Eissa^b^*, Khaled M. Darwish^g^

**Supplementary data**


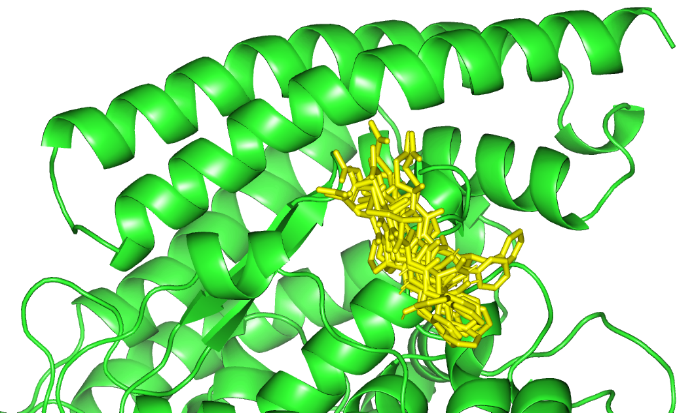


**Fig. S1: Analysis of RMSD trajectories for alacepril-*h*ACE2 protein complex as well as extracted frames at regular time intervals throughout the additional all-atom MD simulation.** The RMSD values are represented relative to backbone and versus MD simulation time in nanoseconds.
